# Fibrin-bearing microparticles: marker of thrombo-embolic events in pancreatic and colorectal cancers

**DOI:** 10.18632/oncotarget.22128

**Published:** 2017-10-26

**Authors:** Diane Mege, Lydie Crescence, Mehdi Ouaissi, Igor Sielezneff, Regis Guieu, Françoise Dignat-George, Christophe Dubois, Laurence Panicot-Dubois

**Affiliations:** ^1^ Aix Marseille Univ, INSERM UMR-S1076, VRCM, Marseille, France; ^2^ Department of digestive surgery, Timone University Hospital, Marseille, France; ^3^ Aix Marseille Univ, UMR MD2, Laboratory of Biochemistry, Timone University Hospital, Marseille, France; ^4^ Laboratory of Hematology, Conception University Hospital, Marseille, France

**Keywords:** microparticle, pancreatic cancer, colorectal cancer, thrombo-embolic events, D-dimers

## Abstract

**Background:**

Microparticles (MPs) are plasma membrane-derived extracellular vesicles present in the bloodstream. We have described a specific signature of MPs, called microparticulosome, in colorectal (CRC) and pancreatic (PC) cancers. We observed that levels of fibrin-bearing MPs were significantly increased in patients suffering from PC and CRC in comparison with control groups. Here, we hypothesised that fibrin-MPs may constitute a relevant biomarker of thrombosis associated with cancer. The objective was to compare the microparticulosome signature between patients presenting with thrombo-embolic event and those without.

**Methods:**

Patients with CRC and PC were prospectively included and divided in those with thrombo-embolic events (Group A) and those without (Group B).

MPs were analyzed by flow cytometer, combining the analysis of Annexin V-positive with characterization of their origin and determination of their procoagulant activities. D-dimer levels were measured in the same samples.

**Results:**

We included 118 patients, divided in 19 patients with thrombo embolic event and 99 patients without. Fibrin-bearing MPs levels were significantly higher in presence of thrombo-embolic events, contrary to D-dimers levels. Fibrin-bearing MPs were more frequently produced by erythrocytes, endothelial cells or Ep-CAM+cells than platelets or leukocytes. Overall survival was shorter in case of thrombo-embolic events than without. The most frequent genes expressed by MPs derived from PC or CRC were implicated in metastatic diffusion of tumor cells, drug resistance, coagulation and inflammation.

**Conclusion:**

Circulating MPs, particularly fibrin-bearing MPs, could be used as a new biomarker to predict cancer-associated thrombo-embolic events and poor survival.

## INTRODUCTION

Cancer-associated venous thromboembolism (VTE) causes significant morbidity and mortality in patients, given that it constitutes the second most common of death, after the progression of the cancer itself. Cancer-associated VTE was firstly described in 1823 by Jean-Baptiste Bouillard and, in 1865 by Armand Trousseau [1]. Defined as deep vein thrombosis and/or pulmonary embolism, VTE occurs more frequently in case of pancreatic ((PC) 5.3%-26%) or brain (1.6-26%) cancers [2]. Many series have reported the poor prognosis of PC in presence of VTE [3-6].

Many risk factors for cancer-associated VTE have been identified, such as patient characteristics (history of VTE, obesity, immobilization), tumor characteristics (location, metastatic), anti-cancer treatments, blood cells (platelet, leukocyte, neutrophil extracellular traps), and hypercoagulable state (thrombin-antithrombin complexes, prothrombin fragment, factor VIII, D dimer, soluble P-selectin, Tissue Factor (TF), microparticles (MPs)). Particularly, Khorana *et al* demonstrated that the occurrence of VTE in PC seems to be correlated with the plasma levels of soluble activated Tissue Factor (TF) [7].

The MPs are heterogeneous extracellular vesicles (0.1-1.0 μm), produced by budding and fission of the plasma membrane from many eukaryotic cells in response to cellular activation (inflammation, cancer) or apoptosis, and express on their surface anionic phospholipids (mainly phosphatidylserine (PS)), cellular origin antigens, and different effectors [8, 9]. They are known to be involved in procoagulant and fibrinolytic activities, vascular remodeling, or neoangiogenesis, through such effectors on their surface as TF and PS, plasminogen activators, inflammatory cytokines, or vascular endothelial growth factor, respectively. In case of cancer, different reports have identified circulating MPs as a main source of TF responsible for thrombosis. These procoagulant MPs may originate from cancer cells themselves and participate not only in the procoagulant state by generating thrombin and fibrin but may also be actively involved in the tumor behaviour and formation of metastasis [10, 11]. To date, different reports have studied the use of TF-bearing MPs as a biomarker of thrombosis in cancer [12]. However, the results of these studies are contradictory with increased levels of TF-bearing MPs in presence of PC-associated VTE [13, 14] and increased procoagulant activity of cancer-associated MPs, in absence of VTE [15-19], or in case of cancer-associated VTE [13, 14, 20-22].

Recently, we described a MPs signature, named the “microparticulosome”, in colorectal cancer (CRC) and PC, compared to inflammatory bowel or pancreatic diseases and healthy subjects [23]. In this study, a significant increase in fibrin-bearing MPs in PC patients was observed compared to the other groups, in accordance with the higher risk of thrombosis in this cancer. However, no significant variation of TF-bearing MPs was noted between groups [23].

Therefore, based on our results previously described and, as the final product of the coagulation cascade is fibrin, we consider that fibrin-bearing MPs may constitute an efficient marker to evaluate the procoagulant activity of cancer-derived MPs.

The aim of the present study was thus to compare the microparticulosome between patients presenting with thrombo-embolic event associated with CRC and PC, and those without thrombo-embolic event.

## RESULTS

### Study population with or without thrombo-embolic event

We included 118 patients presenting with CRC and PC, divided as following:

- In Group A (thrombo-embolic event), 19 patients including 10 CRC and 9 PC. In 14 patients, it was a venous thrombo-embolism (deep vein thrombosis n=7, pulmonary embolism n=7). In the last 5 patients, it was an arterial thrombo-embolic event, with cerebral vascular accident (n=4) and thrombosis of the femoral artery (n=1).

- In Group B (without thrombo-embolic event), 99 patients including 74 CRC and 25 PC.

There was no significant difference between groups regarding to the demographic data and the stage of the cancers (Table 1).

**Table 1:** Demographic data of the 118 patients presenting with colorectal or pancreatic cancer, according to the occurrence or not of a thrombo-embolic event

	Group A	Group B	
	Thrombo-embolic event	No thrombo embolic event	
n	19	99	*P value*
Gender			0.79
Male	12 (63) ^(a)^	67 (68)	
Female	7 (37)	31 (32)	
Age (years)	69 [54–89] ^(b)^	71 [29–93]	0.32
**Colorectal cancer**	10 (53)	74 (75)	
TNM stage ^(c)^			
0	-	6 (8)	0.89
I	2 (20)	17 (23)	
II	3 (30)	22 (30)	
III	3 (30)	17 (23)	
IV	2 (20)	12 (16)	
**Pancreatic cancer**	9 (47)	25 (25)	
TNM stage			
I	-	1 (4)	0.19
II	7 (78)	12 (48)	
III	-	2 (8)	
IV	1 (11)	7 (28)	
NA	1 (11)	3 (12)	

^(a)^: number (percentage); ^(b)^: median (range); ^(c)^: TNM, i.e. Tumor Node Metastasis, classification includes stages I and II for localized tumor, III for locally advanced and IV for metastatic tumor; NA: not available; p<0.05 is considered as significant.

### Comparison of MPs levels

**Figure 1:**
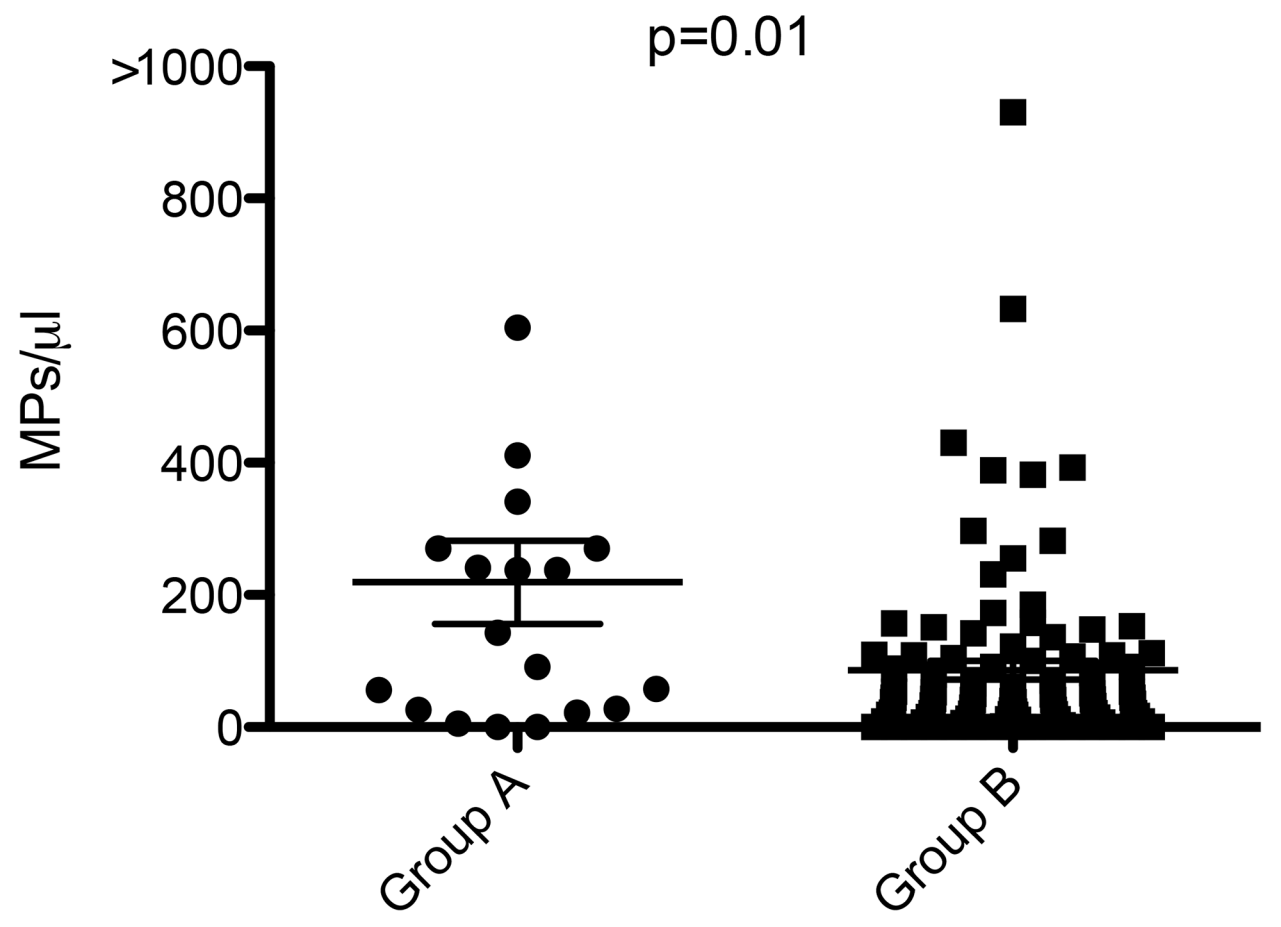
Levels of fibrin-bearing microparticles in case of thrombo embolic event (Group A) and without (Group B), in MPs/ μL

**Figure 2:**
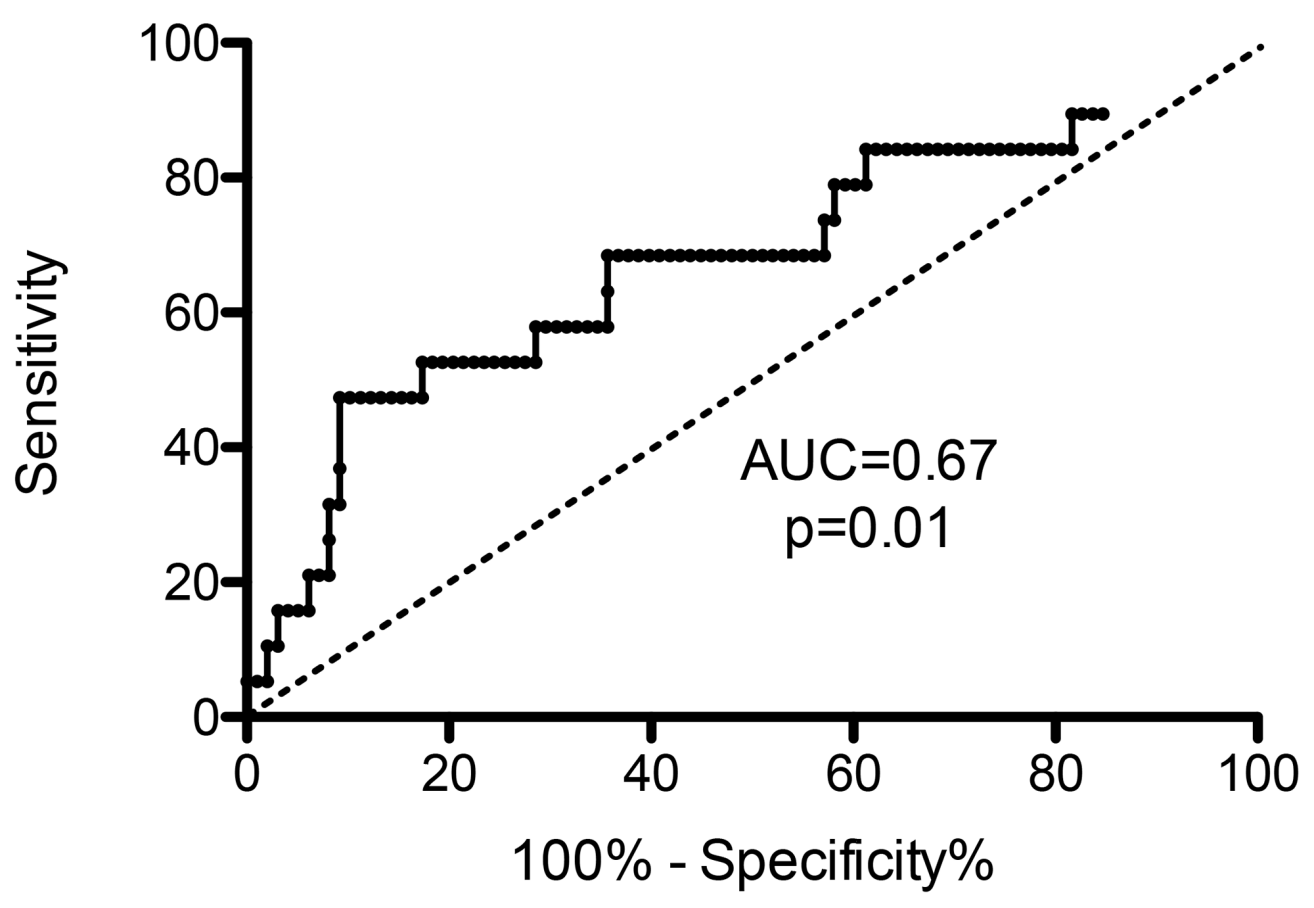
Receiving Operator Characteristics curve and Area under the Curve (AUC) for Fibrin-bearing MPs levels

Total MPs were higher in Group A (8989 [1157-2,119^*^10^6^] MPs/ μL) than in Group B (5866 [865–355459] MPs/ μL, p=0.17), without significant difference. There was no significant difference of MPs levels regarding to the different subpopulations of MPs (*vs* PMPs, EMPs, EryMPs, LeuMPs) (Table 2). TF bearing MPs levels were similar between groups (107 [0–1164] MPs/ μL *versus* 94 [0–3980] MPs/ μL p=0.48), whereas Fibrin bearing MPs levels were significantly higher in Group A (143 [0–1125] MPs/ μL) than in Group B (40 [0–930] MPs/ μL, p=0.01) (Figure 1). The Area Under Curve (AUC) for fibrin bearing MPs was 0.67 (p=0.01), demonstrating a good performance of this parameter in prediction of thromboembolic event (Figure 2). The threshold value with highest intrinsic characteristics (Sensibility = 68%, Specificity = 64%) was equal to 55 Fibrin-bearing MPs/ μL. The different tumor MPs levels were significantly lower in Group A than in Group B.

**Table 2:** Microparticulosome signature based on a color code: high (red) and low (green) levels of MPs (MPs/ μL of plasma), according to the occurrence or not of a thrombo-embolic event

	Group A	Group B	
	Thrombo-embolic event	No thrombo embolic event	
n	19	99	*P value*
Total MPs	**8989**^(a)^ **[1157–2119000]** **(100%)**	**5866** **[865–355459]** **(100%)**	0.17
Subpopulations			
PMPs	**5021** **[110–32515]** **(56%)**^(b)^	**3543** **[288–45959]** **(61%)**	0.28
EMPs	**16** **[1.1-1507]** **(0.2%)**	**3** **[0.6-2340]** **(0.05%)**	0.17
EryMPs	**126** **[0–6641]** **(1%)**	**260** **[0–13026]** **(4%)**	0.65
LeuMPs	**3** **[0–44]** **(0.03%)**	**5** **[0–2972]** **(0.08%)**	0.43
Procoagulant MPs			
TF positive	**107** **[0–1164]** **(1%)**	**94** **[0–3980]** **(2%)**	0.48
Fibrin positive	**143** **[0–1125]** **(2%)**	**40** **[0–930]** **(0.7%)**	**0.01**
Tumor MPs			
PODOPLANIN positive	**37** **[0–4060]** **(0.4%)**	**49** **[0–6456]** **(0.9%)**	0.24
MUC1 positive	**0** **[0–57]** **(0.07%)**	**8** **[0–6673]** **(0%)**	**0.002**
CEA positive	**0** **[0–284]** **(0%)**	**21** **[0–4497]** **(0.4%)**	**<0.0001**
CA19-9 positive	**0** **[0–786]** **(0%)**	**31** **[0–4337]** **(0.6%)**	**0.01**

^(a)^ median (range); ^(b)^ percentage of MPs’ subtypes out of median total MPs; p<0.05 is considered as significant; Total MPs = Annexin V+ MPs; PMPs = platelet-derived MPs (Annexin V+/CD41+); EMPs = endothelial-derived MPs (Annexin V+/CD31+/CD41-);

EryMPs = erythrocyte-derived MPs (Annexin V+/ CD235a+); LeuMPs = leukocyte-derived MPs (Annexin V+/CD11b+/CD235a-); TF positive = Annexin V+/TF+;

Fibrin positive = Annexin V+/Fib+; MUC positive = AnnexinV+/MUC+; CEA positive = AnnexinV+/CEA+; CA 19-9 positive = Annexin +/CA19-9 +.

When we compared the occurrence of thrombo embolic event according to the type of cancer, we observed the same tendency for the different MPs without significant difference, probably due to the small size samples. In CRC and PC, total MPs, PMPs, EMPs, and Fibrin-bearing MPs levels were higher in presence of thromboembolic events than without (Table 3). In CRC, tumor MPs such as MUC1-, ACE- and CA19-9 bearing MPs, were significantly less observed in case of thrombo embolic event (0 [0–57], 0 [0–23] and 0 [0–0] MPs/ μL respectively) than without (15 [0–6673] MPs/ μL, p=0.02; 20 [0–1850] MPs/ μL, p=0.002; and 21 [0–4337] MPs/ μL, p=0.0009, respectively). Taken together these results indicate that fibrin-bearing MPs levels are an efficient marker of cancer-associated thrombo-embolic event.

**Table 3:** Microparticulosome signature based on a color code: high (red) and low (green) levels of MPs (MPs/ μL of plasma), according to the occurrence or not of a thrombo-embolic event, in colorectal and pancreatic cancers

	Colorectal cancer			Pancreatic cancer		
	Thrombo-embolic event	No thrombo embolic event		Thrombo-embolic event	No thrombo embolic event	
n	10	74	*P value*	9	25	*P value*
Total MPs	**7532** ^(a)^ **[1157–16942]** **(100%)**	**5540** **[865–53956]** **(100%)**	0.58	**11920** **[1855–2119000]** **(100%)**	**6544** **[1679–355459]** **(100%)**	0.39
Subpopulations						
PMPs	**4293** **[856–11775]** **(57%)**^(b)^	**3527** **[822–45959]** **(64%)**	0.69	**5131** **[110–32515]** **(43%)**	**3558** **[288–44275]** **(57%)**	0.39
EMPs	**8** **[1.1-29]** **(0.1%)**	**2** **[0.6-836]** **(0.04%)**	0.52	**102** **[1.3-1507]** **(0.9%)**	**45** **[1–2340]** **(0.7%)**	0.33
EryMPs	**113** **[0–6641]** **(1.5%)**	**264** **[0–13026]** **(5%)**	0.40	**448** **[0–3832]** **(4%)**	**207** **[14–8155]** **(3%)**	0.78
LeuMPs	**3** **[0–15]** **(0.03%)**	**4** **[0–792]** **(0.07%)**	0.14	**20** **[0–44]** **(0.2%)**	**8** **[0–2972]** **(0.1%)**	0.75
Procoagulant MPs						
TF positive	**107** **[0–967]** **(1%)**	**81** **[0–3980]** **(1%)**	0.62	**93** **[0–1164]** **(0.8%)**	**126** **[0–1268]** **(2%)**	0.86
Fibrin positive	**101** **[0–1125]** **(1%)**	**40** **[0–633]** **(0.7%)**	0.15	**238** **[0–411]** **(2%)**	**41** **[0–930]** **(0.6%)**	0.08
Tumor MPs						
Podoplanin positive	**20** **[0–98]** **(0.3%)**	**40** **[0–6456]** **(0.7%)**	0.26	**96** **[0–4060]** **(0.8%)**	**85** **[0–988]** **(1%)**	0.5
MUC1 positive	**0** **[0–57]** **(0%)**	**15** **[0–6673]** **(0.3%)**	**0.02**	**0** **[0–11]** **(0%)**	**0** **[0–985]** **(0%)**	0.1
CEA positive	**0** **[0–23]** **(0%)**	**20** **[0–1850]** **(0.4%)**	**0.002**	**0** **[0–284]** **(0%)**	**25** **[0–4497]** **(0%)**	0.06
CA19-9 positive	**0** **[0–0]** **(0%)**	**21** **[0–4337]** **(0.4%)**	**0.0009**	**0** **[0–786]** **(0%)**	**46** **[0–777]** **(0.7%)**	0.64

^(a)^ median (range); ^(b)^ percentage of MPs’ subtypes out of median total MPs; p<0.05 is considered as significant; Total MPs = Annexin V+ MPs; PMPs = platelet-derived MPs (Annexin V+/CD41+); EMPs = endothelial-derived MPs (Annexin V+/CD31+/CD41-);

EryMPs = erythrocyte-derived MPs (Annexin V+/ CD235a+); LeuMPs = leukocyte-derived MPs (Annexin V+/CD11b+/CD235a-); TF positive = Annexin V+/TF+;

Fibrin positive = Annexin V+/Fib+; MUC positive = AnnexinV+/MUC+; CEA positive = AnnexinV+/CEA+; CA 19-9 positive = Annexin +/CA19-9 +.

### Origin of Fibrin+ MPs

In PC samples, fibrin-bearing MPs derived more frequently from erythrocytes (102 [0–15813] MPs/ μL), endothelial cells (61 [0–967] MPs/ μL) or Ep-CAM+ cells (48 [0–15793] MPs/ μL) than platelets (5 [0–16562] MPs/ μL) or leucocytes (2 [0–6756] MPs/ μL; p<0.0001).

### D-dimer levels

Median D-dimer levels were higher in Group A (1170 [192–13050] ng/mL) than Group B (551 [54–4745] ng/mL), but this difference was not significant (p=0.1). According to the type of cancer, there was no significant difference in median D-dimer levels in case of thrombo-embolic event or not: 1170 [237 -2817] vs 564 [54–4686] ng/mL (p= 0.1) in CRC, and 1031 [192–13050] vs 378 [56–4745] ng/mL (p=0.5) in PC.

### Overall survival and thrombo embolic event

**Figure 3:**
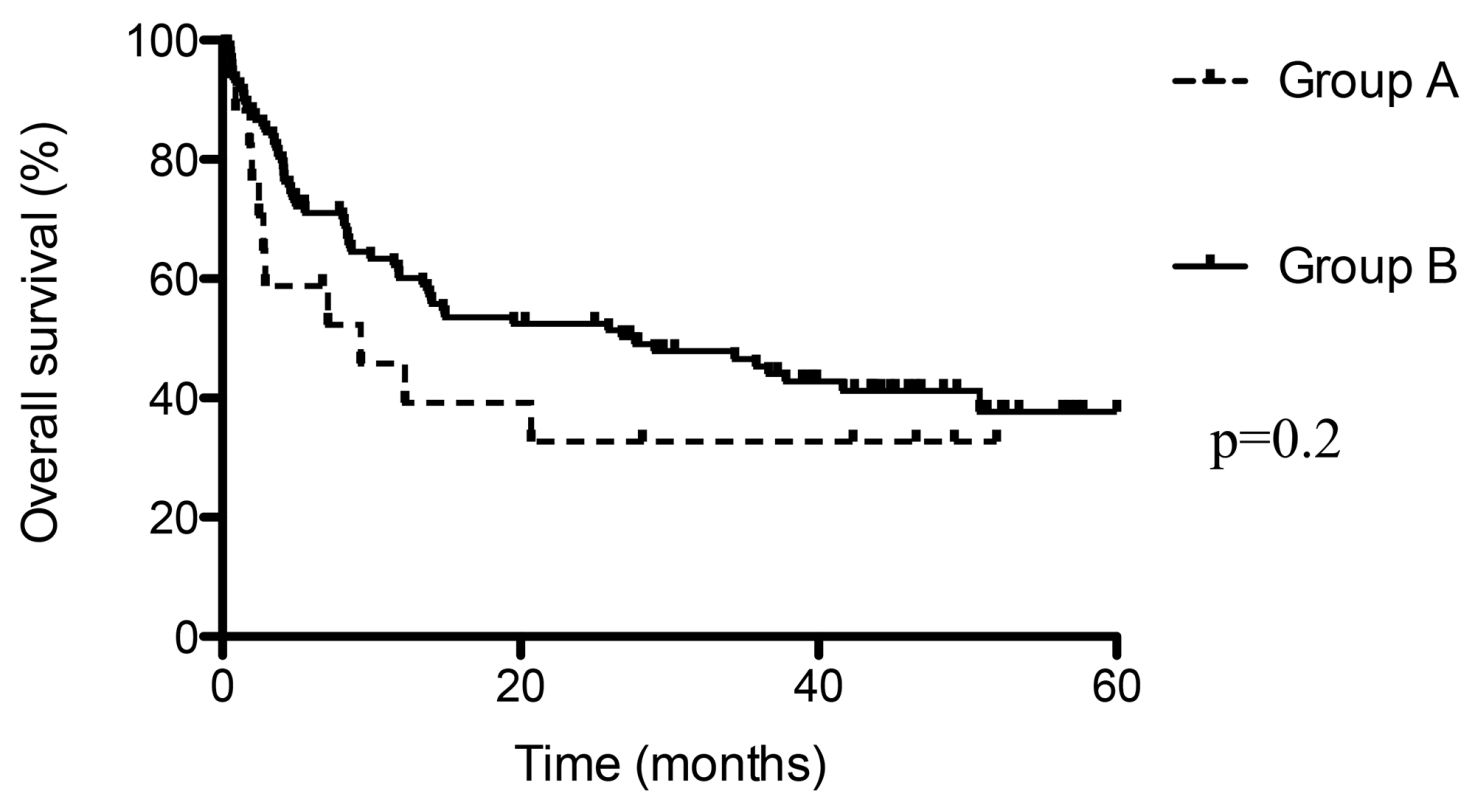
Overall survival in case of thrombo embolic event (Group A) and without (Group B)

**Figure 4:**
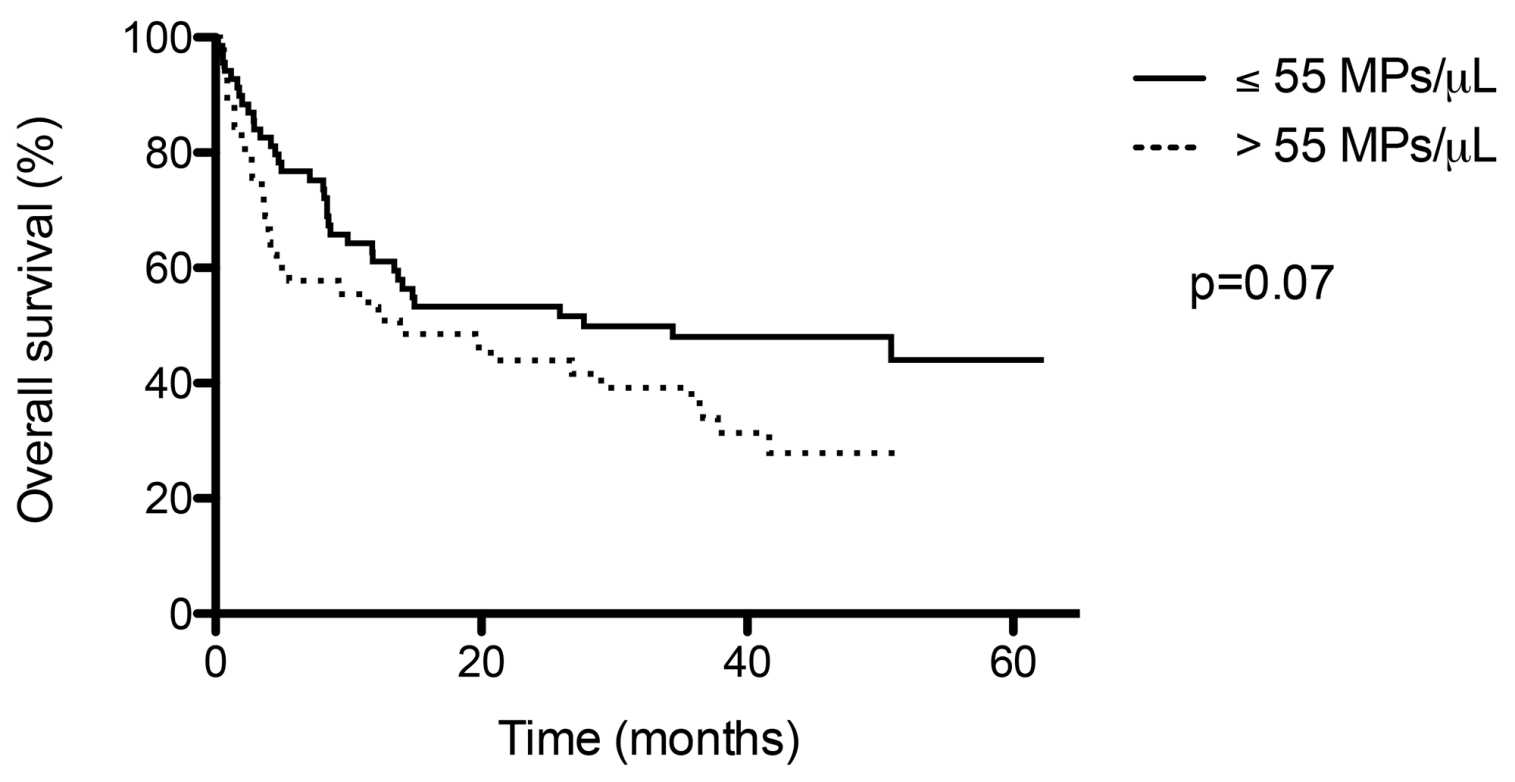
Overall survival according to threshold value of Fibrin-bearing MPs levels (55 / μL)

Median overall survival was shorter in presence of thrombo embolic event (9 months) than without (28 months), but the difference was not statistically significant (p=0.2; Figure 3). This result indicates that thrombo-embolic event associated with cancer could be associated with a poor prognosis. Moreover, we have compared overall survival according to the threshold value of 55 Fibrin-bearing MPs/ μL (Figure 4): median survival was shorter in with levels > 55 MPs/ μL (14 months) than ≤ 55 MPs/ μL (28 months), without reach significance (p=0.07).

### Analysis of RNA profile PC- and CRC-derived MPs

In PC-derived MPs, the most frequently expressed genes were ANXA5 (delta CT=8), ITGB1 (delta CT=9), ADRB2 (delta CT=10), ANXA1 (delta CT=10), HPGD (delta CT=11) and NFKB1 (deltaCT=10).

Similarly, the most frequently expressed genes in CRC-derived MPswere ANXA5 (delta CT=8), ITGB1 (delta CT=10), ANXA1 (delta CT=13), NFKB1 (delta CT=13), MAPK14 (delta Cycle Threshold (CT) =12) and MAPK8 (delta CT=13).

These genes are known to be implicated in proliferation (MAPK8), metastatic diffusion of tumor cells (ITGB1), drug resistance (ANXA1), coagulation (ANXA3, ANXA5) or inflammation (HPGD, NFKB1).

## DISCUSSION

We have reported that circulating MPs levels varied in presence of thrombo-embolic event associated with CRC or PC. Particularly, fibrin-bearing MPs levels were significantly higher in presence of thrombo-embolic event associated with CRC or PC than without. Overall survival was shorter in case of thrombo-embolic event than without.

We did not observe any variation of MPs-TF levels in presence of thrombo-embolic event, neither between different pathological groups in our microparticulosome [23]. Conflicting results about MPs-TF levels are reported in the literature. [13, 14, 19, 26, 27]. This is probably due to lack of standard assay for measuring TF expression and activity on MPs, between immunohistochemical TF staining, enzyme-linked immunosorbent assay, MPs-TF activity assays, and flow-cytometry based methods. Furthermore, an associated explanation may be the quiescent state of the TF on the MPs. Following an injury, TF-expressing cells will accumulate at the site of injury leading to the activation of the blood coagulation cascade. The mechanisms involved in the activation of TF are still unclear. One hypothesis consists in the activation of TF by the protein disulfide isomerase (PDI). PDI is the most abundant thiol isomerase, present in the endoplasmic reticulum, with oxydoreductase functions, forming or cleaving disulphide bonds. The hypothesis is thus that the mechanism of TF de-encryption (*vs*, activation) involves oxidation of 2 free sulphydryls and formation of a disulfide bond, and PDI may be the enzyme that catalyses the formation of that disulfide-bond [28, 29]. Last, for few authors, procoagulant activity of MPs is mainly due to the PS at their surface, as illustrated by higher inhibition of coagulation in presence of Annexin V or Lactadherin, which bind to PS, than in presence of anti-TF antibody [15, 16, 18].

As levels of circulating TF-bearing MPs seem not to be an excellent reflect of procoagulant activity of MPs for the different previous reasons, we have considered that, as the final product of the coagulation cascade is fibrin, the levels of fibrin-positive MPs may directly correlate with the activation state of the blood coagulation cascade. To our knowledge, we are thus the first to evaluate the procoagulant activity of MPs by quantifying the level of fibrin-bearing MPs. Indeed, in the current study, fibrin-bearing MPs levels were significantly higher in presence of thrombo-embolic event associated with CRC or PC. Moreover, in our “microparticulosome”, we have observed significant increased fibrin-bearing MPs levels in the PC group compared to the other groups, which is in accordance with the higher risk of thrombo-embolic event in PC than in CRC [23]. In a recent case series, we reported the diagnosis of a breast cancer in a woman with polymyalgia rheumatic, thanks to elevated level of fibrin-bearing MPs [30]. In these different studies, we have used a standardized protocol based on citrated tubes, strict conditions for storage and preparation, adding of anticoagulant (*i.e* prostacyclin) and fixation of the sample (*i.e* formaldehyde), in order to avoid secondary activation of coagulation after blood collection. For these reasons, fibrin-bearing MPs levels may be an excellent reflect of the procoagulant activity of MPs from blood circulation, and not a product of *in vitro* coagulation. Ep-CAM is a membrane glycoprotein known to be overexpressed in cancers, such as PC [31], breast or lung [32] cancer. After confirmation of the Ep-CAM expression on PC associated MPs, we have observed that the origin of fibrin-bearing MPs is more frequently erythrocytes, endothelial cells or tumour cells (Ep-CAM+) than platelets or leukocytes. This result is in accordance with the implication of MPs in cancer associated thrombo-embolic events.

Some authors have demonstrated the prognosis impact of D-dimer levels on poor survival in ovarian cancer [33], renal cell carcinoma [34] or melanoma [35]. We did not report a significant difference in median D-dimer levels in case of thrombo-embolism event, in our overall study population and neither in CRC or PC subgroups.

Besides to the level of fibrin-bearing MPs, we observed a decrease of MUC1-bearing MPs in case of thrombo-embolic event, whereas two series reported increased levels of MUC1-bearing MPs in presence of VTE [13, 22]. However, our median levels of MUC1-bearing MPs were very low (from 0 [0–57] to 8 [0–6673] MPs/ μL), contrary to Woei-A-Jin *et al* (from 0 [0–221] to 194 [27–392] MPs/ μL) [13]. Although anti-MUC1 antibodies were different between our series (anti-MUC1-647, Novocastra, Newcastle, UK) and the two others (anti-MUC1-FITC, Biomeda, Foster City, CA, USA), which could explain these different identification levels, the difference was probably due to an under-expression of MUC1 by CRC and PC in our study, compared to the two others.

On the other hand, these two series reported also a correlation between elevated procoagulant activity of MPs and shorter overall survival [13, 22]. Indeed, Tesselaar *et al* observed significant shorter median overall survival in case of elevated MPs-TF activity, and in case of VTE associated with PC or breast cancer (2 months) than without VTE (4.5 and 13 months, respectively, p=0.002) [22]. Sherida *et al* noted that, in case of local PC, median overall survival was a significantly shorter in patients with high tumour-TF expression than in those with low tumour-TF expression (12.7 *vs* 25.8 months, p=0.008). The difference in survival was not significant for patients with (locally) advanced disease [13]. In our series, we have also reported that overall survival was shorter in case of thrombo-embolic event than without (median survival 9 *vs* 28 months, p=0.2), but this difference was not significant. Median overall survival was also shorter in case elevated levels of fibrin-bearing MPs (> 55 MPs/ μL: 14 vs 28 months, p=0.07).

Finally, we have showed that among their most frequent expressed genes, PC- and CRC-derived MPs expressed 4 identical genes (ITGB1, ANXA1, ANXA5, NFKB1). These genes are known to be implicated in metastatic diffusion of tumor cells, drug resistance, coagulation or inflammation. These results are in accordance with our previous results suggesting the implication of MPs in cancer-associated thrombo-embolic events. They suggest also the implication of MPs in tumor growth and inflammation. Further analyses are required to confirm these result in patients’ cohort, and compare gene expression levels with occurrence of metastasis, thrombo-embolic event or poor survival.

The main limit of this series is its small sample size, and thus its lake of statistical power, which can explain the absence of significant difference for the separate comparisons of CRC and PC, and for the overall survival. However, there was no bias in analysis and interpretation of data, because preparation and analysis of circulating MPs was performed according to a standardized protocol [36]. The AUC for fibrin-bearing MPs demonstrated the good performance of this parameter in prediction of thromboembolic event (0.67; p=0.01). Moreover, we couldn’t perform RNA profile analyses on our patients’ cohort, because the samples volumes were insufficient, and genetic analyses required specific consent from patients that we didn’t ask when we started our study.

In conclusion, in our study, circulating fibrin-bearing microparticles were more frequently observed in the presence of cancer-associated thrombo-embolic events than D-dimer. High levels of fibrin-bearing microparticles were associated with poor survival. Most of fibrin-bearing microparticles seemed to be produced by erythrocytes, endothelial cells or tumour cells (Ep-CAM+). The most frequent genes expressed by MPs derived from pancreatic or colorectal cancer were implicated in metastatic diffusion of tumor cells, drug resistance, coagulation or inflammation. We thus suggest that circulating microparticles, particularly fibrin-bearing microparticles, could be used as a new biomarker to predict cancer-associated thrombo-embolic events and poor survival. Further larger studies are obviously required to confirm these results.

## MATERIALS AND METHODS

### Study population

We prospectively included all the patients who underwent surgery for CRC and PC in the Digestive Surgical Department of Timone’s Hospital (Marseille, France) between 2009 and 2013. All the individuals signed an institutional review board-approved consent form. Agreement reference of UMR-1076 tissue collection was DC- 2013-1815. Patients under 18 years of age, without informed consent, undergoing surgery for benign colorectal or pancreatic diseases, *i.e*, inflammatory bowel diseases or chronic pancreatitis, and those without pathological diagnosis of malignancy were excluded.

Collected data were demographic (age, sex), clinical (medical and surgical history, TNM stages of PC and CRC and preoperative chemoradiotherapy), laboratory (hemogram, levels of CEA and CA19-9). The occurrence of a thrombo-embolic event, such as venous (deep venous thrombosis, pulmonary embolism) or arterial (cerebral or myocardial infarct) thromboembolism, during the postoperative course or the follow-up, was assessed. The diagnosis of deep venous thrombosis was confirmed by duplex ultrasonography while high-probability ventilation-perfusion lung scan and/or positive angiography were required to establish the diagnosis of pulmonary embolism. Computed tomography scan or magnetic resonance imaging were used to confirm the presence of intra-abdominal or pelvic vein thrombosis, or arterial cerebral infarct. Myocardial infarct was diagnosed in presence of elevated Troponin’s level and coronarography. The following thrombotic events were excluded from the analysis: anyone before blood sample, vascular access-induced thrombosis, superficial thrombophlebitis, thrombosis related to direct extension or compression by malignant tumor and thrombosis occurring in the setting of disseminated intravascular coagulation.

Two groups of patients were thus constituted:

- Group A: patients who presented with thrombo-embolic event

- Group B: patients without thrombo-embolic event.

### Platelet-poor plasma (PPP) containing MPs in human blood

Venous fasted blood samples were obtained the day of the surgery, just before surgery. They were collected in citrated tubes, stored in vertical position and room temperature, and then prepared within two hours following the drawing, in order to avoid coagulation after blood collection, as described by Lacroix *et al* [24]. Citrated blood was centrifuged at 200 g for 15 minutes to obtain the platelet-rich plasma (PRP). Platelet Poor Plasma (PPP) was obtained after centrifugation of PRP at 1100 g for 15 minutes, with prostacyclin (PGI2) added to prevent platelet activation. PPP was then centrifuged at 7000 g for 3 minutes. Aliquots were snap-frozen in liquid nitrogen and stored at -80°C until the measurements were performed.

### Flow cytometric analysis of PPP samples

The circulating MPs contained in the PPP samples were analyzed using a Gallios flow cytometer, as previously described by Robert *et al* [25]. The flow cytometry instrument settings and MP gating were performed with Megamix beads. The MP cellular origin was detected with a combination of Annexin V and specific antibodies against cells of origin. Procoagulant MPs were determined using a combination of Annexin V and antibodies directed against TF and fibrin, the final product of the coagulation cascade. The fibrin antibody was a generous gift from Pr Furie (Harvard Medical School, Boston). These antibodies and their isotype controls were labeled in-house with an Alexa Fluor labeling kit. Tumor MPs were characterized by the detection of MPs (Annexin V positive) expressing tumor markers on their surface, such as Mucine1 (Muc1), Podoplanin, CEA and CA19-9. To find the origin of fibrin-bearing MPs, we have firstly confirmed the expression of Ep-CAM on MPs derived from PC cell lines (BxPC-3 and Capan-2); we have then characterized MPs derived from PC using a combination of MPs cellular origin antigens or Ep-CAM with fibrin.

Matched isotype control antibodies were labeled in-house with an Alexa fluor labeling kit. Each specific antibody was matched with an isotype control antibody labeled with an appropriate fluorescent dye. The MPs labeling was based on a 30-minute incubation of patient PPP (30 μL) with Annexin V and appropriate antibodies; 300 μL of calcium buffer was then added to improve the binding of Annexin V to PS. After a 3-minute incubation, 100 μL of formaldehyde (16%) was added to fix the sample (and thus avoid coagulation), and 30 μL of CytoCount beads was added; theses beads were necessary to determine the concentration of MPs in PPP. The concentrations are expressed as the number of MPs/ μL of PPP. The samples and associated controls were measured within an hour using a Gallios flow cytometer. The sample analyses were performed with Kaluza software [24]. We used a standardized flow cytometry protocol, based on fluorescent calibrated beads (0.1, 0.3, 0.5, and 0.9 μm) called Megamix (BioCytex, Marseille, France). Forward scatter threshold of discriminator value and photomultiplier tube voltage were regularly adjusted, in order to reduce background noise and thus detect with more accuracy the different MPs subpopulations (with size from 0.2 to 1 μm), as reported by Robert *et al* [25].

### D-dimer level measurement

D-dimer levels (expressed in ng/mL) were measured from PPP samples, using Stratus^®^ CS Analyzer (Siemens, Germany).

### Analysis of RNA profile PC- and CRC-derived MPs

MPs were obtained from human PC (BxPC-3) and CRC (HT29) cell lines (ATCC, Manassas, VA). Total RNA for quantitative RT-PCR (RT-qPCR) and microarray analyses was extracted from BxPC-3 or HT29 derived MPs using the Trizol/chloroform solution (Thermo Fisher Scientific, Waltham, MA) according to the manufacturer’s instructions. The RNA was reverse transcribed using Superscript IV Reverse Transcriptase, outRNase and oligo(dT) primers (Thermo Fisher Scientific, Waltham, MA). RT-qPCR reactions were performed using a Taqman qPCR kit (Taqman Thermo Fisher Scientific, Waltham, MA) and analyzed by StepOne real-time PCR detection system (Life Technologies, Carlsbad, CA). The RNA microarray experiments were performed with the TaqMan^®^ Gene Signature Arrays using a Human Inflammation expression array (Life Technologies, Carlsbad, CA), which contains 96 gene types. To study gene expression changes between HT29 and BxPC-3 MPs, 94 genes were selected based on an inflammation pathway product (Life Technologies, Carlsbad, CA). All genes were expressed in CT). We reported the 6 most frequently expressed genes normalized to 18S (expressed in delta CT) in the two cell lines.

### Statistical analysis

The qualitative and categorical variables are expressed as percentages and the median with a range. The MPs levels, expressed as MPs/ μL, and D-dimer levels, expressed in ng/mL, are compared between groups using the Student t. All the tests were two-tailed, and differences were considered significant at p<0.05. In order to evaluate intrinsic characteristics of MPs levels, Receiving Operator Characteristics curve was performed with calculation of the AUC. All the analyses were performed using the GraphPad software (Graphpad Software Inc., San Diego, California, USA).

## Abbreviations

VTE: venous thromboembolism
MPs: microparticles
TF: tissue factor
PS: phosphatidylserine
PC: pancreatic cancer
CRC: colorectal cancer
AUC: area under the curve
PMPs: platelet-derived MPs
EMPs: endothelial cell-derived MPs
PDI: protein disulfide isomerase
MUC1: mucin 1
CEA: carcinoembryonic antigen
CA19-9: carbohydrate antigen 19-9
PRP: platelet-rich plasma
PPP: Platelet Poor Plasma
PGI2: prostacyclin
RNA: ribonucleic acid
RT-PCR: reverse transcription polymerase chain reaction
CT: cycle threshold
